# Six steps to standardize the surgical approach for ingrown toenail^☆^^☆☆^

**DOI:** 10.1016/j.abd.2020.04.012

**Published:** 2020-11-18

**Authors:** Han Ma

**Affiliations:** Department of Dermatology, Guangdong Provincial Key, Laboratory of Biomedical Imaging, Fifth Affiliated Hospital, Sun Yat-sen University, Zhuhai, Guangdong Province, China

**Keywords:** Nails, ingrown, Orthopedics, Surgery, plastic

## Abstract

**Background:**

Dermatologists don’t have a good knowledge of the surgical treatment for ingrown toenails and there is no consensus on which is the best approach.

**Objective:**

To develop an easy and effective surgical approach to solve the problem of ingrown toenails.

**Methods:**

We identified 67 patients with ingrown toenails in varying degrees of severity which were treated with the standardized approach.

**Results:**

All the patients had a completely recovery from the disease and none complained about the cosmetic result.

**Study limitations:**

The number of cases is limited.

**Conclusion:**

The standardized surgical approach is easily learned and very effective. The recurrence rate is lower than with other treatments.

## Introduction

Ingrown toenail is a very common disease that causes pain and disability. There are many different surgical strategies to address this condition, which rely on two main approaches: narrowing the nail plate or debulking soft tissues.^1^ This study presents an improvement in the surgical technique, by combining the two approaches, and performing the standardized six steps to ensure a higher cure rate and appropriate aesthetic effect. This research was approved by the Ethics Committee of the Fifth Affiliated Hospital, Sun Yat-sen University, under protocol number K148-1.

## Methods

**Figure 1:**
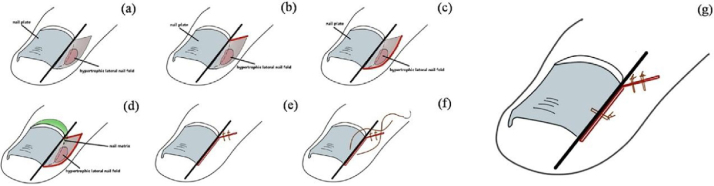
Standardized six steps for ingrown toenail.

The study enrolled 67 patients with ingrown toenail in varying degrees of severity between January 2018 and September 2019. The procedure was performed through the following standardized six steps (Fig. 1A–G): (A) A longitudinal resection line was identified according to the part of ingrown nail that needed to be removed; (B) from the junction point between the margin of eponychium and the longitudinal resection line, a 45-degree angle excision was performed; (C) an arc incision was made along the lateral nail fold, assuring that the range included the hypertrophic granulation tissue; (D) the ingrown nail plate was cut along the longitudinal line and the hypertrophic tissue and corresponding part of nail matrix were completely removed; (E) the proximal lateral nail fold was sutured, using two or more Nylon 4/0 sutures; (F) in order to fixate the nail margin on the toe/finger, one or more Nylon 2/0 sutures were performed as needed; (G) the immediate appearance of the toe/fingernail after the operation.

## Results

**Figure 2:**
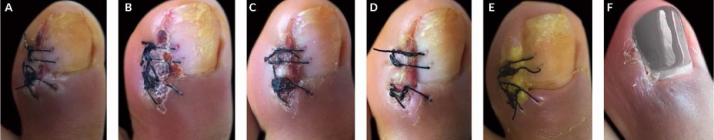
Post-operative healing process. (A) 3^rd^ day, (B) 4^th^ day, (C) 5^th^ day, (D) 6^th^ day, (E) 14^th^ day, (F) 4^th^ week.

**Figure 3:**
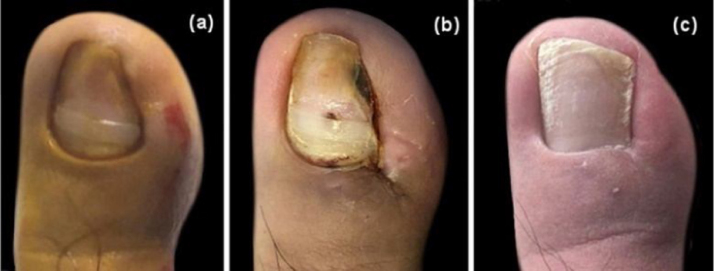
Cosmetic result. (A), Preoperative image. (B), After stitch removal on the 14^th^day. (C), Follow-up after one year.

The healing process lasted from three to four weeks, approximately, and sutures were removed on the 14^th^ postoperative day (Fig. 2). Fifteen of 67 patients received the treatment for the bilateral toenails. Almost all the patients experienced postsurgical pain, but only five required non-steroid anti-inflammatory drugs (NSAIDs). All the cases received the management with water-filtered infrared-A (wIRA) and only three severe cases presented local infection, requiring antibiotics. The appearance of the nail was nearly unchanged, and no patients complained about the cosmetic results (Fig. 3).

## Discussion

Ingrown nails seriously impact the daily life of patients, most of whom underwent a long and repeated course of the condition before being referred to a dermatologist. In fact, a large number of dermatologists are unfamiliar with the surgical technique and there is no consensus on which is the best operation to treat ingrown toenails. The recurrence rate for surgical approaches is between 1.7% and 27%.^2^ In contrast, the present technique was used to treat 67 patients, and had no recurrence observed after six months to one year of follow-up.

**Figure 4:**
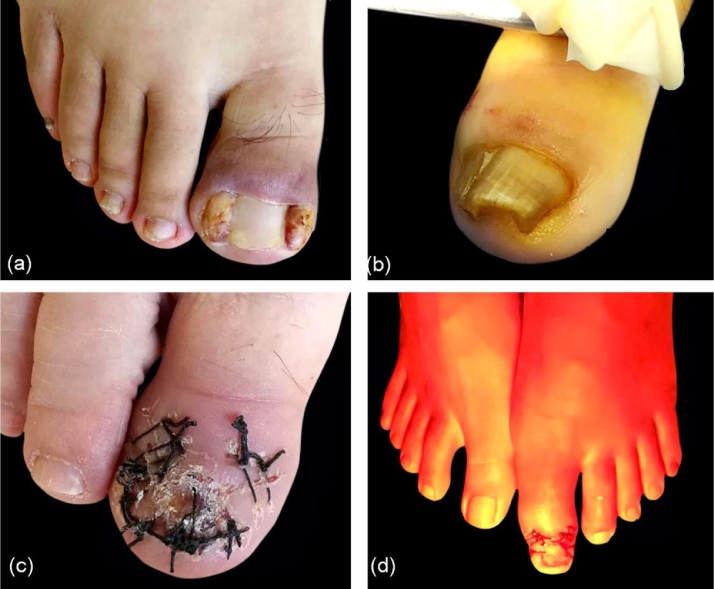
Key points. (A), Large masses in triangular regions. (B), Thick nail plate. (C), Remove the entire nail plate before the operation. (D), Management with water-filtered infrared-A (wIRA).

Subsequently, the key points about the procedure are listed. (1) Step 4 is the most important procedure to avoid the recurrence. The author’s experience is that it is enough to cut off the nail root tissues and see the white phalanx. In order to minimize the risk of recurrence, a small wedge-shaped resection inward is usually performed at the end point of the nail plate, which does not affect the nail appearance. (2) Sometimes the disease course was too long and a large mass can be observed in the triangular region, so it might be difficult to suture the nail plate and surrounding tissues in step 6, if the resection was too wide and left an ample space beside the nail, as shown in Fig. 4A. It is better to keep 0.5 cm width; even if a small amount of necrotic tissue is left behind, it would be absorbed gradually. (3) If the nail plate is too thick (Fig. 4B) to cross-section easily, the entire plate should be removed before the procedure (Fig. 4C). (4) In order to better protect the nail bed and decrease the risk of postsurgical infection, a “Y” dressing and water-filtered infrared-A (wIRA) were used after the operation (Fig. 4D). They were effective techniques, as suggested by the literature.^3^, ^4^

## Conclusion

The author introduces an effective and simple surgical method to treat ingrown toenails. The recurrence rate is lower than the other methods.

## Financial support

None declared.

## Author contribution

Han MA: Approval of the final version of the manuscript; design and planning of the study; drafting and editing of the manuscript; collection, analysis, and interpretation of data; effective participation in research orientation; intellectual participation in the propaedeutic and/or therapeutic conduct of the studied cases; critical review of the literature; critical review of the manuscript.

## Conflicts of interest

None declared.
